# Exploring the intersection of epigenetics, DNA repair, and immunology from studies of ICF syndrome, an inborn error of immunity

**DOI:** 10.3389/fimmu.2024.1405022

**Published:** 2024-05-10

**Authors:** Motoko Unoki

**Affiliations:** Department of Human Genetics, School of International Health, Graduate School of Medicine, The University of Tokyo, Tokyo, Japan

**Keywords:** ICF syndrome, epigenetics, DNA methylation, DNA repair, NHEJ, class-switch recombination, hypoglobulinemia, immunodeficiency

## Abstract

Immunodeficiency, centromeric instability, and facial anomalies (ICF) syndrome, a rare autosomal recessive disorder, manifests with hypoglobulinemia and chromosomal instability accompanied by DNA hypomethylation. Pathological variants in the *DNMT3B*, *ZBTB24*, *CDCA7*, or *HELLS* genes underlie its etiology. Activated lymphocytes from patients often display distinctive multiradial chromosomes fused via pericentromeric regions. Recent studies have provided deeper insights into how pathological variants in ICF-related proteins cause DNA hypomethylation and chromosome instability. However, the understanding of the molecular pathogenesis underlying immunodeficiency is still in its nascent stages. In the past half-decade, the roles of CDCA7, HELLS, and ZBTB24 in classical non-homologous end joining during double-strand DNA break repair and immunoglobulin class-switch recombination (CSR) have been unveiled. Nevertheless, given the decreased all classes of immunoglobulins in most patients, CSR deficiency alone cannot fully account for the immunodeficiency. The latest finding showing dysregulation of immunoglobulin signaling may provide a clue to understanding the immunodeficiency mechanism. While less common, a subgroup of patients exhibits T-cell abnormalities alongside B-cell anomalies, including reduced regulatory T-cells and increased effector memory T- and follicular helper T-cells. The dysregulation of immunoglobulin signaling in B-cells, the imbalance in T-cell subsets, and/or satellite RNA-mediated activation of innate immune response potentially explain autoimmune manifestations in a subset of patients. These findings emphasize the pivotal roles of ICF-related proteins in both B- and T-cell functions. ICF syndrome studies have illuminated many fundamental mechanisms. Further investigations will certainly continue to unveil additional mechanisms and their interplay.

## 1 Introduction

Immunodeficiency, centromeric instability, and facial anomalies (ICF) syndrome is a rare autosomal recessive disorder characterized by reduced immunoglobulin levels in the serum and recurrent, often fatal, respiratory and gastrointestinal infections of bacteria, viruses, fungi, and/or parasites (1, 2). ICF patients possess naïve B-cells but lack memory B-cells and plasma cells in their peripheral blood. Centromeric instability manifests as stretched heterochromatin, chromosome breaks, and multiradial configurations involving the pericentromeric regions of chromosomes 1, 9, and 16, which are composed of highly repetitive satellite-2 or -3 (hSATII or hSATIII) repeats and exhibit hypomethylation in patients’ cells. While chromosomal rearrangements are primarily observed in T-cells, only a subset of ICF patients demonstrates apparent T-cell immunodeficiency. Additionally, ICF patients display facial anomalies and neurologic defects across a spectrum of severity.

To date, five genes have been reported to be mutated in ICF patients: *DNA methyltransferase 3B* (*DNMT3B*), *zinc finger and BTB domain containing 24* (*ZBTB24*), *cell division cycle associated 7* (*CDCA7*), *helicase lymphoid specific* (*HELLS*, also known as *LSH*), and *Ubiquitin-like containing PHD and RING finger domains 1* (*UHRF1*, also known as *ICBP90* or *Np95*) in type-1 (ICF1), -2 (ICF2), -3 (ICF3), -4 (ICF4), and atypical ICF patients, respectively (3, 4). Briefly, DNMT3B functions as a *de novo* DNA methyltransferase, ZBTB24 serves as a transcriptional factor whose targets include *CDCA7*, and CDCA7 and HELLS proteins constitute a chromatin remodeling complex. Because of the absence of immunodeficiency in the atypical case with hypomorphic variants in *UHRF1*, an essential factor for maintenance of DNA methylation alongside DNMT1 (5), we focus on ICF1–4 in this mini-review.

## 2 Chromosome instability in ICF patients

Recent studies have unveiled that DNA hypomethylation patterns observed in patients’ cells can be broadly categorized into three types based on their underlying mechanisms. The first pattern arises from pathogenic variants in *DNMT3B*, causing impairment in DNA methylation establishment during post-implantation development, resulting in hypomethylation at distinct regions represented by pericentromeric and subtelomeric regions. The second pattern results from pathogenic variants in *ZBTB24*, *CDCA7*, and *HELLS*, disrupting replication-uncoupled maintenance DNA methylation. Consequently, specific regions, where chromatin remodeling by the CDCA7/HELLS complex is required, undergo hypomethylation. These hypomethylated regions typically exhibit characteristics of the B genomic compartment, heterochromatin, and/or late replicating regions (6, 7), encompassing pericentromeric and centromeric regions, but not including subteromeric regions. The third pattern arises from pathogenic variants in *UHRF1*, which could attenuate the efficiency of both replication-coupled and -uncoupled maintenance DNA methylation, resulting in a distinct hypomethylation pattern. The hypomethylated regions include pericentromeric, centromeric, and subtelomeric regions. Detailed molecular mechanisms regarding DNA methylation maintenance are described elsewhere (3).

Among the five ICF types, pericentromeric regions composed of hSATII or hSATIII repeats are commonly hypomethylated. Utilizing ICF model human embryonic kidney (HEK) 293 cells, in which ICF causative genes were knocked out via genome editing, we have demonstrated abnormal transcription of hSATII mRNA from the hypomethylated regions. This could cause unphysiological R-loops, leading to DNA double-strand breaks (DSBs) (8). This mechanism sheds light on the occurrence of chromosome instability in ICF patients’ cells. Notably, lymphoblastoid cells derived from ICF patients and the ICF model cells exhibit slower growth rates compared to control cells with an increase of apoptotic cells (9). The enlarged nucleus observed in the ICF model cells, along with a significant increase in centrosome numbers and size, likely due to excessive DSBs, suggests that these cells may undergo cell cycle slippages from G2 into G1 due to prolonged G2. While the ICF model cells manage to withstand these abnormalities and survive, primary cells may be more sensitive and undergo apoptosis under such severe stresses.

## 3 B-cell deficiency in ICF patients

**Figure 1:**
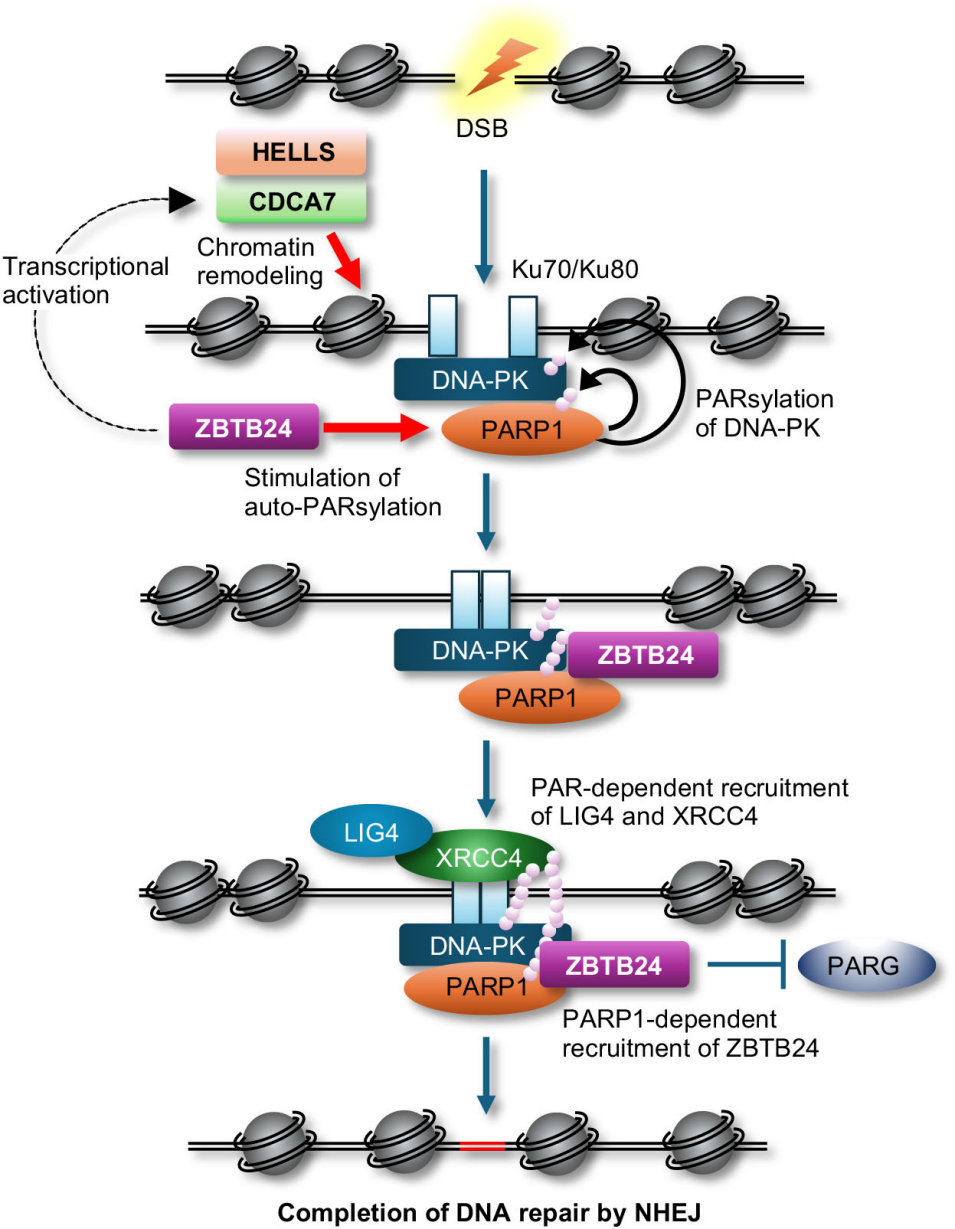
A possible model of c-NHEJ during DSB repair. The CDCA7 and HELLS complex assists Ku proteins and DNA-PK in accessing DSB sites possibly by their chromatin remodeling activity, while ZBTB24 enhances auto-PARsylation of PARP1, which stimulates kinase activity of DNA-PK via PARsylation. ZBTB24 recognizes the PAR chains on PARP1, protects the PAR chains from degradation by PARG, resulting in the facilitation of NHEJ by PAR-dependent assembly of the LIG4/XRCC4 complex at DSB sites.

By conducting a comprehensive analysis to identify interacting proteins of CDCA7, we discovered that CDCA7 interacts with Ku80 and DNA-dependent protein kinase (DNA-PK), both of which are involved in classical non-homologous end joining (c-NHEJ) in DSB repair, V(D)J recombination, and class-switch recombination (CSR) (10). Furthermore, we revealed that CDCA7 and HELLS assist Ku80 in accessing DSB sites and facilitate NHEJ possibly through their chromatin remodeling activity (9). Subsequently, Helfricht et al. demonstrated that ZBTB24 can directly facilitate c-NHEJ. They found that ZBTB24 stimulates auto-poly(ADP-ribosyl)ation of poly(ADP-ribose) polymerase 1 (PARP1) and subsequently recognizes the PAR chains on PARP1 to localize at DSB sites (11). This localization prevents degradation of PAR chains by PAR glycohydrolase (PARG), resulting in the facilitation of NHEJ via the PAR-dependent assembly of the DNA ligase 4 (LIG4)/X-ray repair cross-complementing 4 (XRCC4) complex at the DSB sites. Although PARP1 can be involved in c-NHEJ via stimulation of DNA-PK kinase activity by PARsylation and alternative NHEJ (12), their findings suggest that ZBTB24 promotes c-NHEJ. Hence, ZBTB24 can facilitate c-NHEJ directly via the interaction with PARP1 and indirectly through transcriptional activation of *CDCA7* (**Figure 1**).

It has been demonstrated that immunoglobulin CSR heavily relies on the c-NHEJ-mediated repair of activation-induced cytidine deaminase (AID)-induced DSBs (13). Recently, He et al. demonstrated that HELLS facilitates NHEJ in CSR in B-cells using the hematopoietic cell lineage-specific *Hells* conditional KO (cKO) mice (14). While serum IgM levels remain unchanged, IgG levels are significantly decreased in these mice, suggesting that HELLS aids in CSR from IgM to IgG in mice. Furthermore, they showed that HELLS is not required for the initiation of CSR, including induction of AID, chromatin accessibility at switch regions, and formation of DSBs, but is required for the completion of recombination. Around the same time, Helfricht et al. implicated the involvement of human ZBTB24 in the final stage of CSR by demonstrating that ICF2 patients possess almost unchanged numbers of unswitched memory B-cells but possess decreased numbers of switched B-cells in their peripheral blood (11). They also showed that V(D)J recombination and AID induction are intact in B-cells from ICF2 patients.

**Figure 2:**
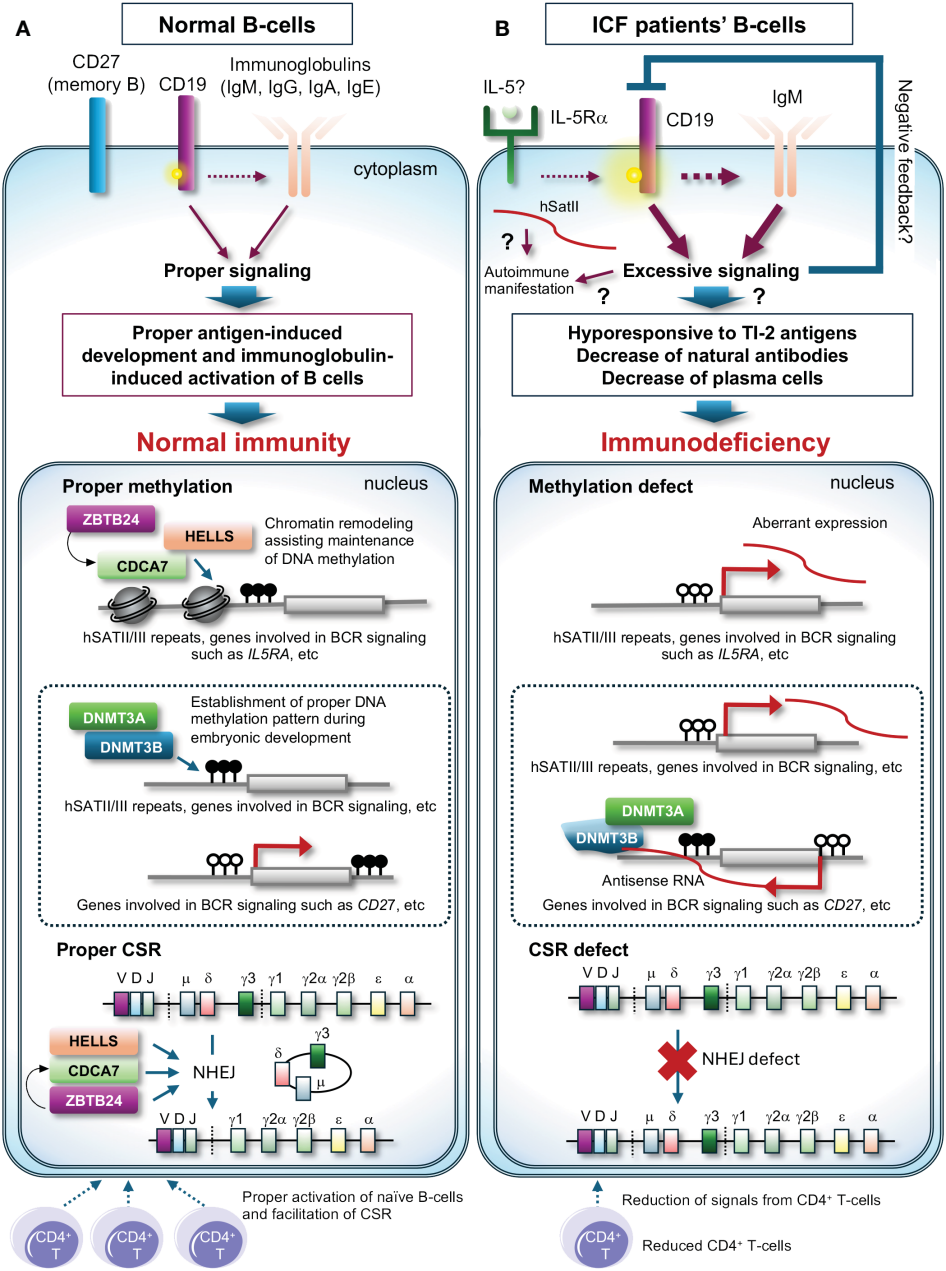
A possible molecular pathogenesis behind immunodeficiency of ICF patients. **(A)** In normal B-cells, class-switch recombination (CSR) properly occurs upon signals from CD4^+^ T-cells with the assistance of the CDCA7/HELLS complex and ZBTB24, and the promoter region of genes involved in immunoglobulin signaling possibly including the *IL5RA* promoter is likely highly methylated and maintained the status with the assistance of the CDCA7/HELLS complex, resulting in proper expression levels of these genes. In this status, phosphorylation levels of CD19 are normal, resulting in proper antigen-induced development and immunoglobulin-induced activation of B-cells. The proper methylation patterns are established by DNMT3B and/or DNMT3A during embryonic development. **(B)** In ICF patients’ B-cells, CSR is deficient, and the promoter regions of genes involved in immunoglobulin signaling possibly including the *IL5RA* promoter are hypomethylated, resulting in induced expression of these genes. High expression of IL-5Rα could facilitate phosphorylation of CD19 and the downstream signaling. This may induce negative feedback and/or cause hypo-responsiveness to T-independent type 2 (TI-2) antigens, a reduction of natural antibodies, and a decrease in plasma cells, resulting in immunodeficiency. In ICF1 cells, DNMT3B defects could cause aberrant methylation, resulting in aberrant expression of genes possibly including *CD27*, a memory B-cell marker. Dysregulation of T cells may exacerbate immunodeficiency. White and black circles on the genes indicate unmethylated and methylated cytosines, respectively. ? indicates potential pathways contributing to the outcomes.

Here, the remaining largest question is that levels of all classes of immunoglobulins (IgM, IgG, and IgA) are frequently decreased in all types of ICF patients, which cannot be solely attributed to CSR deficiency. The latest findings by Ying et al. may provide a clue (15). They reported that plasma cells, along with all classes of immunoglobulins, are reduced in the hematopoietic cell lineage-specific *Zbtb24* cKO mice, possibly due to increased phosphorylation levels of CD19 in B-cells, known to establish intrinsic B-cell signaling threshold. The *Zbtb24* cKO mice are hypo- and hyper-responsive to T-independent type 2 (TI-2) and T-dependent (TD) antigens, respectively. They also discovered that the expression of *interleukin 5 receptor subunit alpha* (*Il5ra*) is elevated in *Zbtb24*-deficient B-cells accompanied by drastic hypomethylation of its promoter, and increased IL-5Rα enhances phosphorylation of CD19. Their findings suggest that decreased natural antibodies due to impaired responses to TI-2 antigens including repetitive surface structures of encapsulated bacteria are likely the major cause of immunodeficiency, and abnormal responses to TD antigens may also contribute to ICF phenotype (**Figure 2**). Two decades ago, Blanco-Betancourt et al. hypothesized that negative selection could be impaired in ICF B-cells because ICF naïve B-cells bear potentially autoreactive long heavy chain variable regions complementarity determining region 3 (V_H_CDR3), composed of full-length *D_H_* gene segments (16). Because human CD19 transgenic mice demonstrate elevated levels of autoantibodies alongside displaying enhanced immunoglobulin signaling (17), CD19 activation could explain why ICF B-cells often carry autoreactive immunoglobulins. In summary, while HELLS and ZBTB24 are crucial for CSR, the dysregulation of immunoglobulin signaling in ICF B-cells may have a more significant impact on B-cell abnormalities, potentially masking CSR deficiency in many ICF cases.

Another remaining question is how pathogenic variants in *DNMT3B*, which encodes a *de novo* DNA methyltransferase, affect immunoglobulin levels in ICF1 patients. An early attempt to clarify the molecular pathogenesis using murine models carrying pathogenic variants found in ICF1 patients has yielded a result slightly different from the expectation, as the models do not exhibit B-cell deficiency (18). A more recent comprehensive study by Gatto et al. demonstrated that many genes involved in the immunoglobulin signaling pathway are differentially expressed in ICF1 patients’ immortalized lymphoblast cell lines (19). Intriguingly, in the cell lines, expression of *CD27*, a memory B-cell marker, is decreased alongside its promoter hyper-methylation possibly via recruitment of the DNMT3B/DNMT3A complex on its antisense transcript (19). They also detected increased alternative splicing events of CD45, a regulator of immunoglobulin and T-cell receptor signaling, in the cell lines (19). These transcriptional alterations mediated via aberrant DNA methylation and/or RNA binding ability of DNMT3B or its interacting proteins may explain decreased memory B-cells and plasma cells in ICF1 patients’ peripheral blood (**Figure 2**), although this pioneering work needs to be confirmed using patients’ primary cells in the future.

## 4 T-cell deficiency and autoimmune manifestation in ICF patients: Possible involvement of regulatory T (Treg) cells in ICF pathogenesis

Since some ICF patients suffer from recurrent viral and/or fungal infections mostly with bacterial infections, ICF patients have been suspected of having concomitant T-cell immunodeficiency (1, 2). Although the molecular mechanism remains elusive, a subset of ICF patients exhibit lymphopenia and/or an inverted CD4^+^/CD8^+^ ratio due to the reduction of CD4^+^ T-cells, which potentially hampers activation of naïve B-cells and CSR (**Figure 2**). It is also demonstrated that the number of apoptotic lymphocytes in mitogen-stimulated cells was significantly higher in patients than in controls (20). This could be attributed to the frequent occurrence of abnormal chromosomes in activated T-cells (1).

In addition to these abnormalities, recent studies have shown that Treg cells, which suppress immune response, and recent thymic emigrant T-cells (RTEs), which maintain T-cell diversity in the periphery, were decreased in many ICF patients, while effector memory CD4^+^ T-cells (CD4^+^T_EM_), which augments the immune response, and circulating follicular helper T-cells (cT_FH_), which are increased in the periphery of patients with autoimmune diseases, were increased in the majority of ICF patients (2, 21, 22). While less common, approximately 10 to 20% of patients show autoimmune manifestations. The manifestations in ICF patients can be explained by activation of the immunoglobulin signaling in B-cells accompanied possibly by CD19 activation (see chapter 3) but reduced total numbers of Treg cells and/or increased CD4^+^T_EM_ cells and cT_FH_ cells in patients may also contribute to these manifestations (23, 24). Moreover, it has been reported that enhanced expression of pericentromeric repeats hSATII RNA in senescent and cancer cells traps CCCTC-binding factor (CTCF), resulting in the alteration of chromatin structure and subsequent activation of inflammatory genes (25). In addition to our finding, of which expression of hSATII is increased in ICF model cells (8), the innate immune response is activated by sensors of cytosolic RNA, which recognize aberrantly expressed satellite repeats from hypomethylated pericentromeric regions, in *Zbtb24* knockout zebrafish (26). Hence, the increased expression of pericentromeric repeats can also contribute to the autoimmune manifestation (**Figure 2**).

## 5 ICF murine models

The phenotype related to immune cells in murine ICF models slightly differs from ICF patients. The ICF1 murine models demonstrate increased apoptosis of thymocytes after birth, while B-cells remain normal (18). Although an ICF2 murine model lacking *Zbtb24* specifically in the hematopoietic lineage displays similar phenotypes of ICF2 patients (see chapter 3), *Zbtb24* null mice exhibit embryonic lethality, which contrasts with ICF2 patients, some of whom carry null variants (15, 27, 28). Similarly, *Hells* null mice exhibit perinatal lethality, despite some ICF4 patients having null variants (29, 30). Another ICF4 murine model reconstituted with bone marrow from hematopoietic lineage*-*specific *Hells* cKO mice shows reduced B-cell numbers and skewed CD4^+^ T cell proportions (14). Considering the phenotypic differences among ICF patients and various murine models, ICF-related proteins could have similar but slightly different roles and/or different compensational proteins in murine and human tissues composed of various cell types, including niche cells in the bone marrow and the thymus. However, these observations highlight the potential involvement of ICF-related proteins in both human and murine B- and T-cell functions.

## 6 Conclusions

ICF studies have been uncovering key cellular regulatory mechanisms, spanning DNA methylation, DNA repair, and immune cell regulation. Since the understanding of immunodeficiency in ICF patients has just started, and facial anomalies and neurologic defects in ICF patients have not been well-studied, ICF studies will continuously contribute to further deepening insights into fundamental life mechanisms.

## 7 Postscript

One recent excellent work by Cousu et al. (31) was accidentally overlooked in the main text. Using B-cell-specific Hells cKO mice, they found that HELLS plays a pivotal role in T-dependent B-cell responses; HELLS deficiency induces the accelerated decay of germinal center (GC) B cells and impairs the generation of high-affinity memory B cells and circulating IgGs. In addition, mutant GC B cells undergo dramatic DNA hypomethylation, leading to the premature upregulation of either memory B cell markers or the transcription factor ATF4, which drives an mTORC1-dependent metabolic program typical of plasma cells. Although CSR is unlikely affected by the absence of HELLS in the mice, as IgM and IgA levels are unchanged, their findings shed light on why IgG levels are frequently decreased in all types of ICF patients. I appreciate Prof. Sébastien Storck (Université Paris Cité) for notifying me of this oversight.

## Author contributions

MU: Writing – original draft, Writing – review & editing.
