# Corrigendum: Exploring the intersection of epigenetics, DNA repair, and immunology from studies of ICF syndrome, an inborn error of immunity

**DOI:** 10.3389/fimmu.2024.1445756

**Published:** 2024-06-28

**Authors:** Motoko Unoki

**Affiliations:** Department of Human Genetics, School of International Health, Graduate School of Medicine, The University of Tokyo, Tokyo, Japan

**Keywords:** ICF syndrome, epigenetics, DNA methylation, DNA repair, NHEJ, class-switch recombination, hypoglobulinemia, immunodeficiency

In the published article, there was an error. The author accidentally overlooked an important recent work by Prof. Sébastien Storck’s group (Université Paris Cité).

A correction has been made to the newly generated section, “Postscript”, which has been inserted after “6 Conclusions” and before “Author Contributions”.

The corrected sentence appears below:

“One recent excellent work by Cousu et al. (31) was accidentally overlooked in the main text. Using B-cell-specific *Hells* cKO mice, they found that HELLS plays a pivotal role in T-dependent B-cell responses; HELLS deficiency induces the accelerated decay of germinal center (GC) B cells and impairs the generation of high-affinity memory B cells and circulating IgGs. In addition, mutant GC B cells undergo dramatic DNA hypomethylation, leading to the premature upregulation of either memory B cell markers or the transcription factor ATF4, which drives an mTORC1-dependent metabolic program typical of plasma cells. Although CSR is unlikely affected by the absence of HELLS in the mice, as IgM and IgA levels are unchanged, their findings shed light on why IgG levels are frequently decreased in all types of ICF patients. I appreciate Prof. Sébastien Storck (Université Paris Cité) for notifying me of this oversight.”

**Missing Citation**

Citation (31) has been added according to the addition of the “Postscript”.

31. Cousu C, Mulot E, Smet AD, Formichetti S, Lecoeuche D, Ren J, et al. Germinal center output is sustained by HELLS-dependent DNA-methylation-maintenance in B cells. Nat. Commun (2023) 14:5695. doi: 10.1038/s41467-023-41317-3

The authors apologize for this error and state that this does not change the scientific conclusions of the article in any way. The original article has been updated.

